# Characterization of extracellular vesicle-associated DNA and proteins derived from organotropic metastatic breast cancer cells

**DOI:** 10.1186/s13046-025-03418-3

**Published:** 2025-05-23

**Authors:** Amélie Nadeau, Thupten Tsering, Mohamed Abdouh, Laura Kienzle, Jenna Cleyle, Lorne Taylor, Noélie Douanne, Kyle Dickinson, Peter M. Siegel, Julia V. Burnier

**Affiliations:** 1 Cancer Research Program, Research Institute of the McGill University Health Centre, Montreal, QC Canada; 2 Department of Pathology, McGill University, Montreal, QC Canada; 3 Centre for Translational Biology, Research Institute of the McGill University Health Centre, Montreal, QC Canada; 4 Rosalind & Morris Goodman Cancer Institute, McGill University, Montreal, QC Canada; 5 Department of Medicine, McGill University, Montreal, QC Canada; 6 Gerald Bronfman Department of Oncology, McGill University, Montreal, QC Canada

**Keywords:** Extracellular vesicles, Metastatic breast cancer, Organotropism, Proteomics, DNA, Biomarkers

## Abstract

**Background:**

While primary breast cancer (BC) is often effectively managed, metastasis remains the primary cause of BC-related fatalities. Gaps remain in our understanding of the mechanisms regulating cancer cell organotropism with predilection to specific organs. Unraveling mediators of site-specific metastasis could enhance early detection and enable more tailored interventions. Liquid biopsy represents an innovative approach in cancer involving the analysis of biological materials such as circulating tumor DNA and tumor-derived extracellular vesicles (EV) found in body fluids like blood or urine. This offers valuable insights for characterizing and monitoring tumor genomes to advance personalized medicine in metastatic cancers.

**Methods:**

We performed in-depth analyses of EV cargo associated with BC metastasis using eight murine cell line models with distinct metastatic potentials and organotropism to the lung, the bone, the liver, and the brain. We characterized the secretome of these cells to identify unique biomarkers specific to metastatic sites.

**Results:**

Small EVs isolated from all cell lines were quantified and validated for established EV markers. Tracking analysis and electron microscopy revealed EV secretion patterns that differed according to cell line. Cell-free (cf)DNA and EV-associated DNA (EV-DNA) were detected from all cell lines with varying concentrations. We detected a *TP53* mutation in both EV-DNA and cfDNA. Mass spectrometry-based proteomics analyses identified 698 EV-associated proteins, which clustered according to metastatic site. This analysis highlighted both common EV signatures and proteins involved in cancer progression and organotropism unique to metastatic cell lines. Among these, 327 significantly differentially enriched proteins were quantified with high confidence levels across BC and metastatic BC cells. We found enrichment of specific integrin receptors in metastatic cancer EVs compared to EVs secreted from non-transformed epithelial cells and matched tumorigenic non-metastatic cells. Pathway analyses revealed that EVs derived from parental cancer cells display a cell adhesion signature and are enriched with proteins involved in cancer signaling pathways.

**Conclusion:**

Taken together, the characterization of EV cargo in a unique model of BC organotropism demonstrated that EV-DNA and EV proteomes were informative of normal and cancer states. This work could help to identify BC biomarkers associated with site-specific metastasis and new therapeutic targets.

**Supplementary Information:**

The online version contains supplementary material available at 10.1186/s13046-025-03418-3.

## Background

Breast cancer (BC) remains a significant global health concern, representing the most diagnosed malignancy and a leading cause of cancer mortality among women worldwide [1]. Globally, almost 2.3 million new BC cases are reported in women each year, accounting for 11.7% of all cancer cases, and is responsible for an estimated 685,000 deaths annually [1]. While incidence rates vary geographically [1], BC is the most common cancer diagnosed among women in Canada, affecting 1 in 8 and the second leading cause of cancer-related death [2, 3]. Despite its high incidence, the mortality rate from BC has been decreasing largely due to advancements in early detection [4] and treatment [5, 6]. Although the 5-year net survival rate is high for BC (89%), it varies by stage at diagnosis and is reduced significantly at 23.2% for stage IV [7]. This prompts the urgent need to develop new approaches to understand and treat this aggressive form of cancer, as well as new biomarkers to monitor disease progression.

Despite advancements in treatment, 20–30% of women initially diagnosed with earlier stages of BC will eventually experience recurrent advanced or fatal metastatic disease. Metastatic BC (MBC) can be detected in about 6% of newly diagnosed cases [8–10]. Common distant metastatic sites include the bone, the liver, the lung, and the brain [11]. The colonization of specific organs by particular subpopulations of cancer cells is not a random process [12], yet gaps remain in our understanding of the mechanisms underlying the predilection of cancer cells for specific sites, also known as organotropism [13]. Understanding the mediators of site-specific metastasis would contribute to earlier diagnosis and tailor better interventions for patients.

Several influential studies on MBC genetics have revealed that very few recurring mutations are unique to metastatic tumors, except for *ESR1* mutations, which are primarily associated with resistance to hormone therapy [14–17]. Compared to primary tumors, metastases appear to harbour slightly increased incidence of well-known oncogenic mutations (such as *TP53*, *PTEN*, and *RB1*), changes in mutational patterns [18], and minor increases in DNA amplifications and deletions [14, 19]. In addition, the incidence of brain metastasis is reported to be higher in those with ERBB2 positive and triple negative BC [20]. Consequently, the aggressive nature of MBC cannot be fully explained by genetic changes alone, highlighting the need for more comprehensive evaluation across multiple ‘omics’ analyses [11]. Moreover, the lack of longitudinal tissue sampling limits real time analysis of disease progression and identification of molecular changes associated with metastasis.

Liquid biopsy represents an innovative and minimally invasive approach involving the analysis of biological material that can be found in body fluids, like the blood [21, 22]. These analytes include circulating tumor cells, cell-free circulating tumor DNA (ctDNA) and tumor-derived extracellular vesicles (EVs), which provide valuable material to characterize and monitor the tumor genome, proteome and lipidome, and their changes over time [23–25]. However, given their low abundance in bodily fluids, better methods to study these analytes are needed, such as in vitro culture systems.

EVs are lipid-bound particles released by various cell types and play major roles in intercellular communication by transporting proteins, nucleic acids, and lipids over short and long distances [26–31]. These vesicles offer stability and protection to their cargo via their lipidic bilayer membrane, shielding their molecular cargo from the extracellular environment [26]. EV uptake by target cells involves multiple pathways [32, 33], and the molecular processes governing EV formation and cargo delivery have been discussed [34–36]. Notably, studies have shown that cancer cells release significantly more small (s)EVs than non-transformed cells, highlighting their potential utility in cancer research [37, 38]. Mounting evidence indicates that EVs harbor selective cargo influencing cancer progression and metastasis [39–42], with roles in organotropism [43] and pre-metastatic niche formation [44, 45]. This positions them as valuable candidates for biomarkers in disease diagnosis, prognosis, and monitoring [46–49]. The proteomic profile of MBC secretome and BC-derived EVs have been reported in an attempt to understand their role in cancer [50, 51]. However, to the best of our knowledge, no studies have characterized the differential expression of proteins between parental and organotropic MBC-derived EVs.

Apart from protein expression, EV-DNA is garnering particular interest for its potential utility as a biomarker that can be isolated through liquid biopsy [52–58], reviewed in [59]. Contributing to this rapidly evolving field, we developed the EV-ADD (evdnadatabase.com), a publicly accessible database of published data on EV-DNA [60]. Emerging evidence suggests that both circulating cell-free DNA (ccfDNA) and EV-DNA can be exploited as non-invasive biomarkers of disease and for molecular genotyping and mutational profiling [59, 61].

In this study, we conducted an in-depth molecular analysis of EVs isolated from murine BC cell lines with distinct metastatic potential and organotropisms to the lung, the bone, the liver, and the brain. Using nanoparticle tracking, mass spectrometry and droplet digital PCR analyses, we compared the EV emission patterns, proteomic cargo and EV-DNA between non-transformed mammary epithelial cells, primary BC and site-specific MBC cell lines. Overall, we characterized and quantified the cargo of EVs in cell models of BC organotropism, shedding light into potential biomarkers for non-invasive liquid biopsy testing and identifying potential mediators of site-specific metastasis that could serve as therapeutic targets for MBC.

## Methods

### Cell culture

We used murine cell models composed of the 4T1 parental cell line and their derivative metastatic cell lines with different organotropism to the lung, the bone, the liver, and the brain. These include non-transformed NMuMG cells, non-metastatic 67NR and metastatic 4T1 parental BC cell lines, lung-MBC (4T1-533, 4T1-537), bone-MBC (4T1-592, 4T1-593), and liver-MBC (4T1-2776, 4T1-2792), and brain-MBC (4T1-BP, 4T1-LM) cell lines [62–68]. The cells were cultured in DMEM (319–007-CL, Wisent, QC, Canada) supplemented with 10 mM HEPES (330–050-EL, Wisent, QC, Canada), 10 µg/ml insulin (4,693,124,001, Roche, Basel, Switzerland), 0.1% 10 U/ml penicillin and 10 µg/ml streptomycin (30–001-CI, Corning, NY, USA), amphotericin B (450–105-QL, Wisent, QC, Canada), and 10% fetal bovine serum (35–077-CV, Corning, NY, USA). Cells were maintained in a humidified incubator at 37°C with 5% CO_2_. All cell lines were tested and negative for mycoplasma (LT07-418, Lonza, Basel, Switzerland).

### EV isolation

Once cells reached 70–80% confluency, they were washed twice with PBS, and fresh media with 10% EV-depleted FBS was added. The FBS was depleted of bovine EVs by ultracentrifugation (Beckman 70 Ti rotor) at 120,000 g for 18 h at 4°C and filtration (0.2 µm). After 24 h of incubation, the cell culture conditioned media (CCM) was collected and processed by a first centrifugation at 500 g for 20 min to remove potential cell contamination. The supernatant was then collected and spun at 2000 g for 20 min to remove potential large cell debris and apoptotic bodies. Next, the supernatant was filtered using a syringe filter (0.2 µm) and stored at −20 °C prior to EV isolation. EVs were isolated from three independent replicates for each cell line with 90 ml of CCM for DNA analysis and 200 ml of CCM for proteomics analysis. The collection of large volumes of CCM required concentration before the separation of EVs from other particles. Hence, the supernatant from previous steps was centrifuged at 4000 g for 20 min using an ultra-centrifugal filter (UFC910024, MilliporeSigma, MA, USA), retaining EVs while discarding many proteins [27]. The concentrated EVs were resuspended in filtered PBS (0.2 µm) and spun at 120,000 g for 70 min at 4 °C. The supernatant was carefully discarded, and the EV pellet was spun with fresh filtered PBS for a second ultracentrifugation at 120,000 g for 70 min at 4°C. The final EV pellet was resuspended in filtered PBS, measured for protein concentration by BCA, and stored at −80°C for further assays.

### Transmission electron microscopy (TEM)

EV preparations were directly coated on formvar carbon grids, fixed with 1% glutaraldehyde in 0.1 M sodium cacodylate buffer for 1 min, and stained with 2% uranyl acetate for 3 min. Formvar grids coated with isolated EVs were recorded using a FEI Tecnai 12 120 kV TEM. The resulting images were captured with the AMT XR-80 C CCD Camera System.

### Scanning electron microscopy (SEM)

Cells were seeded into 6 well plates containing poly-L-lysine 12 mm glass coverslips (72,292–01, Electron Microscopy Sciences, PA, USA) and allowed to grow for 24 h or until cultures reached 70% confluency. Cells were fixed with 2.5% glutaraldehyde in 0.1 M cacodylate buffer for 60 min at 4°C. The cells were then post-fixed in 1% osmium tetroxide (Electron Microscopy Sciences, PA, USA) for 30 min, followed by washing with deionized water three times for 15 min each. Cells on the coverslip were dehydrated with a graded ethyl alcohol (ethanol): dH_2_O series, i.e. 30%, 50%, 70%, 80%, 90%, 95% and 100% (× 2) at room temperature; 10 min for each step. The cover slips were immediately transferred to the Leica Microsystems EM CPD300 for Critical Point Dehydration. To avoid charging, 4 nm platinum was deposited by sputter coating with Leica EM ACE600 sputter coater. Samples were imaged using a Quanta 450 FE-SEM at a high vacuum mode. The imaging mode used was Secondary Electron Detector, with a working distance of 10 mm, at 5 kV, spot 5, 50 µm aperture.

### Nanoparticle tracking analysis (NTA)

Concentrations and size distribution of EVs isolated from 90 ml of CCM were characterized by NTA using the NanoSight NS500 system (Nanosight Ltd., Amesbury, UK). Five sequential 30-s videos were collected and analyzed using the NTA 1.3 software (Malvern Panalytical, Malvern, UK). The average size and concentration of the particles were calculated by integrating the averages of the means from three independent recordings, each obtained from three independent biological replicates.

### DNA isolation

DNA was extracted from EVs isolated from 90 ml of CCM processed as described above. Using the QIAamp DNA Mini Kit (51,304, Qiagen, Hilden, Germany) according to the manufacturer’s instruction, DNA was eluted twice in a total of 50 μl of nuclease-free water for further quantification. cfDNA was recovered from 4 ml of CCM collected as described above. cfDNA was isolated from 4 ml of CCM using the EZ2 automated system with the ccfDNA Kit (954,854, Qiagen, Hilden, Germany) using the protocol according to the manufacturer’s instructions, which is customized to elute DNA in 45 μl of buffer EZE. EV-DNA and cfDNA samples (4 µl) were quantified using the Qubit fluorometer dsDNA high sensitivity kit (Q32854, Invitrogen, MA, USA) according to the manufacturer’s protocol.

### Droplet digital polymerase chain reaction (ddPCR)

All cell-derived EV samples were tested for the presence of DNA mutations using ddPCR. The detection of *Trp53* P31X was carried out using Affinity Plus® Mini probes, with the wild-type probe labeled as 5'HEX™/3'IB®FQ (/5HEX/TA + TCT T + C + T + G + GA GGA/3IABkFQ/) and the mutant probe labeled as 5'FAM™/3'IB®FQ (/56-FAM/AT C + TT + C + T + T GGA + G + GA/31 ABkFQ/). ddPCR amplification was achieved using the forward primer (GCC TGG GAT AAG TGA GAT TCT G) and reverse primer (CTT TCT GCT CTG GGC CTT AC). The annealing temperature was optimized using gradient PCR. ddPCR was performed according to the manufacturer's protocol, using 10 μl 2 × ddPCR Supermix for probes, 900 nM primers (Integrated DNA Technologies, IA, USA), 250 nM probes (FAM/HEX, Affinity Plus qPCR Probes, Integrated DNA Technologies, IA, USA), up to 8 μl of DNA template, and nuclease-free water. For each reaction, the master mix described above with DNA templates were added to the cartridge, followed by 70 μl of Droplet Generation Oil (1,863,005, Bio-Rad Laboratories, CA, USA). Droplets were generated using the QX200 Droplet Generator (Bio-Rad Laboratories, CA, USA). The droplets were transferred to a 96-well plate and sealed with foil (1,814,040, Bio-rad Laboratories, CA, USA). A total of 40 PCR cycles were carried out as follows: 1 × 95°C (10 min), 40x (95°C (30 s), annealing temperature for 60°C (60 s), and 72°C (30 s)) and 1 × 98°C (10 min). The plate was read using the QX200 Droplet Reader (Bio-Rad Laboratories), and data was analyzed with the QuantaSoft software. All samples were performed in duplicate.

### EV protein isolation

EVs and cells were lysed using RIPA buffer supplemented with cOmplete™ Mini Protease Inhibitor Cocktail (11,836,170,001, Sigma-Aldrich, MO, USA) at 4°C for 30 min while vortexed every 10 min. The samples were spun at 13,000 g for 30 min at 4°C. Supernatants containing EV-associated proteins were collected and quantified using the Micro BCA assay (23,235, Thermo Scientific, MA, USA).

### Western blot

To verify the presence of proteins in our preparations, 10 μg of EVs proteins (as per Micro BCA) were separated using 4%−12% precast polyacrylamide gel and transferred onto polyvinylidene fluoride (PVDF) membranes (4,568,094, Bio-Rad Laboratories, CA, USA). Membranes were blocked for 1 h in 5% non-fat dry milk in Tris buffer saline with 0.05% Tween-20 (TBST). Membranes were probed with anti-TSG101 (1:1000) (ab125011, abcam, Cambridge, UK), anti-syntenin (1:1000) (ab19903, abcam, Cambridge, UK), anti-CD81 (1:1000) (ab109201, abcam, Cambridge, UK), anti-calnexin (1:1000) (ab22595, abcam, Cambridge, UK), and anti-albumin (1:1000) (A2228, Sigma-Aldrich, MO, USA). Membranes were washed in TBST and were treated with corresponding horseradish peroxidase-conjugated secondary antibodies anti-rabbit HRP (7074S, Cell Signalling Technology, MA, USA) and anti-mouse HRP (7076P2, Cell Signalling Technology, MA, USA). Blots were developed using ECL prime Western blot detection (GE Healthcare, IL, USA) and visualized using the ChemiDoc™ XRS + System (Bio-Rad Laboratories, CA, USA).

### Mass spectrometry (MS) analysis

EV-associated proteins isolated from 200 ml of CCM were analysed by MS. For each sample, 20 μg of EV proteins were loaded onto a single-stacking gel band to remove lipids, detergents and salts. A single gel band containing all proteins was reduced with DTT, alkylated with iodoacetic acid and digested with trypsin. Extracted peptides were re-solubilized in 0.1% aqueous formic acid and loaded onto a Thermo Acclaim Pepmap (164,946, ThermoFisher Scientific, MA, USA) precolumn and then onto an Acclaim Pepmap Easyspray (164,534, ThermoFisher Scientific, MA, USA) analytical column separation using a Dionex Ultimate 3000 uHPLC at 250 nl/min with a gradient of 2–35% organic (0.1% formic acid in acetonitrile) over 3 h. Peptides were analyzed using a Thermo Orbitrap Fusion mass spectrometer operating at a resolution of 120,000 (FWHM in MS1) with HCD sequencing (15,000 resolution) at top speed for all peptides with a charge of 2+ or greater.

### Protein identification and quantification

The raw data were converted into *.mgf format (Mascot generic format) for searching using the Mascot 2.6.2 search engine (Matrix Science, MA, USA) against mouse protein sequences (Uniprot 2023). False discovery rates at the peptide and protein levels were fixed at 1%. The database search results were loaded onto Scaffold Q+ Scaffold_5.0.1 (Proteome Sciences, Surrey, UK) for statistical treatment and data visualization. To account for variations across samples and conditions, total spectrum counts (TSC) for proteins detected by MS were normalized in Scaffold (protein threshold: 99%; minimum # peptides: 5; peptide threshold: 95%). A one-way ANOVA was applied to the differentially quantified proteins, and *p*-values < 0.05 were considered significant.

### Bioinformatics analysis

Mascot was searched with a fragment ion mass tolerance of 0.100 Da and a parent ion tolerance of 5.0 PPM. Carboxymethyl cysteine was specified in Mascot as a fixed modification. Deamidated of asparagine and glutamine and oxidation of methionine were specified in Mascot as variable modifications.

Criteria for Protein Identification: Scaffold (version Scaffold_5.1.1, Proteome Software Inc., Portland, OR) was used to validate MS/MS based peptide and protein identifications. Peptide identifications were accepted if they could be established at greater than 95.0% probability by the Peptide Prophet algorithm [69] with Scaffold delta-mass correction. Protein identifications were accepted if they could be established at greater than 99.0% probability and contained at least 5 identified peptides. Protein probabilities were assigned by the Protein Prophet algorithm [70]. Proteins that contained similar peptides and could not be differentiated based on MS/MS analysis alone were grouped to satisfy the principles of parsimony.

The quantified EV-associated proteins were examined through Principal Component Analysis (PCA) in MarkerView 1.3.1 (SCIEX).. Their abundance was represented by Venn diagrams constructed using the online tool http://bioinformatics.psb.ugent.be/webtools/Venn/. Heatmap clustering was generated using R (version 4.2.3) with Complexheatmap package [71]. The protein ratios were calculated between the site-specific MBC cell lines (i.e. 4T1-533, 4T1-537, 4T1-592, 4T1-593, 4T1-2776, 4T1-2792, 4T1-BP, 4T1-LM), 4T1 parental cells, non-metastatic 67NR cells and the non-transformed NMuMG cell line. Calculated Z-score was used to plot the heatmaps. The Functional Enrichment Analysis Tool (FunRich) software 3.1.3 (http://www.funrich.org/) was used to compare our data to Vesiclepedia (http://www.microvesicles.org/). The gene ontology (GO) and the Kyoto Encyclopedia of Genes and Genomes (KEGG) pathway analysis were conducted through the Database for Annotation, Visualization and Integrated Discovery (DAVID) functional annotation tools [72, 73].

### Statistical analysis

The data were analyzed using unpaired two-tailed t-tests for comparison between the different groups. A *P*-value < 0.05 was considered statistically significant.

## Results

**Fig. 1:**
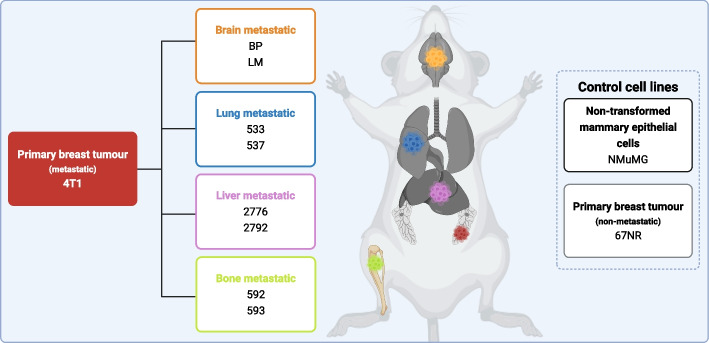
Parental and derivative metastatic cell lines with distinct organotropism. Created in BioRender. Burnier, J. (2025) https://BioRender.com/zhjiic3

To explore the secretome associated with organotropism, we used murine cell models of BC with distinct metastatic potential and organotropism to four metastatic sites (Fig. 1). The 4T1 and 67NR mammary carcinoma cell lines are subpopulations of a single BALB/c/C3H mouse mammary tumor, with 4T1 forming spontaneous mammary carcinoma metastases, whereas 67NR is a cell line that does not leave the primary site [62–64, 74, 75]. The 4T1 cell line can metastasize to distant sites (lung, bone, liver, brain) from the mammary fat pads of immunocompetent mice. In vivo selection of the 4T1 parental cells was used to generate independent and aggressive site-specific MBC populations by previous researchers [62–68]. For the purpose of our research, two different cell lines from each metastatic site were used. Namely, these include the cell lines 4T1-533 and 4T1-537 explanted from lung metastases [65], 4T1-592 and 4T1-593 cell lines explanted from bone metastases [66], 4T1-2776 and 4T1-2792 cell lines explanted from liver-metastases [67], and 4T1-BP (brain parenchyma) and 4T1-LM (leptomeninges) cell lines from brain metastases [68]. In addition, the non-transformed NMuMG (normal murine mammary gland) cell line was cultured and used for comparisons with BC cells. The morphology and cell growth of the cell lines were characterized (Figure S1, Figure S2).

### Cell-derived EVs displayed different emission patterns and morphology across cell models

**Fig. 2:**
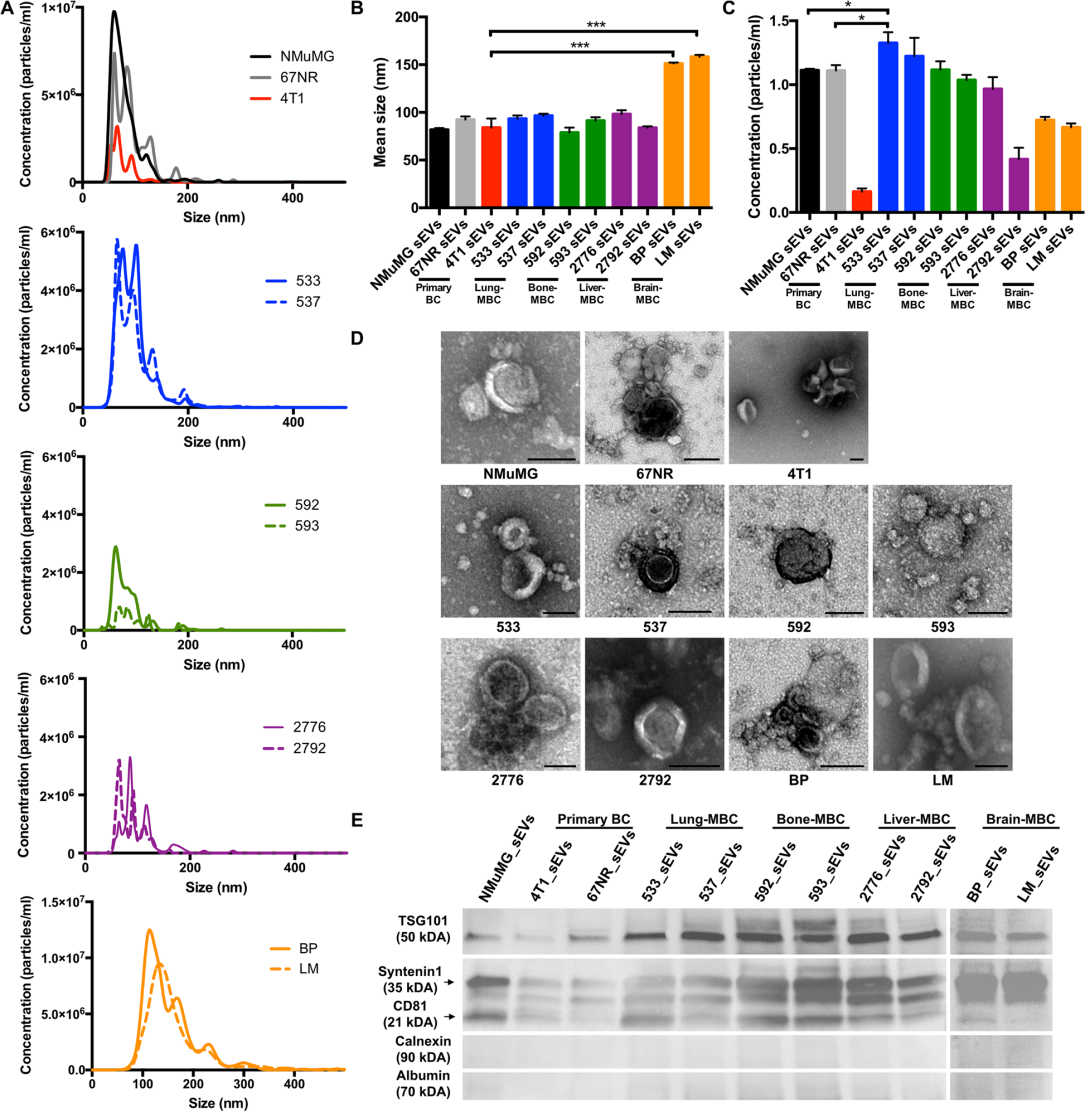
Characterization of EVs derived from cell cultures. **A** Size distribution, **B** average sizes, and (**C**) concentration of EVs as measured by NTA. Data were normalized to cell counts. **D** Micrographs of EV preparations by TEM. Scale bar = 100 nm. **E** Western blot analysis of specific proteins isolated from cell-derived EVs. In B and C, data are presented as mean ± SD (*n* = 3 independent experiments, *P < 0.05 and ***P < 0.001)

To characterize EVs within this organotropism model, we isolated, quantified and characterized EVs physically and phenotypically from all cell lines, with consideration of the Minimal information for studies of extracellular vesicles (MISEV) guidelines [27, 29, 76]. Cell-derived EVs displayed a mean diameter of 100.9 nm across all cell models (Fig. 2A-B), characteristic of small EVs (sEVs) [29]. In contrast to a modest increase observed in other groups, brain-MBC EVs (4T1-BP, 4T1-LM) were significantly larger (154.8 nm) than 4T1 parental EVs (84.2 nm) (*P* = 0.0002) (Fig. 2A-B). These findings suggest that brain-MBC EVs may have unique biophysical properties that could influence their interaction with recipient cells.

The quantification of EVs showed variations in emission among the different cell lines. Lung-MBC (4T1-533, 4T1-537) and cell lines emitted significantly more EVs than non-transformed NMuMG cells and non-metastatic 67NR (Fig. 2C). Notably, 4T1 parental cells emitted significantly less EVs than all other cell lines (Fig. 2C).

Further characterization revealed the presence of round-shaped vesicles, with typical cup shape artefacts formed by desiccated conditions during TEM imaging [29] (Fig. 2D). Western blot analysis confirmed the expression of proteins generally enriched in EVs including cluster of differentiation 81 (CD81), tumor susceptibility gene 101 (TSG101) and syntenin-1, while they were negative for the expression of calnexin and albumin, supporting the purity of the EV preparations (Fig. 2E, Figure S3).

**Fig. 3:**
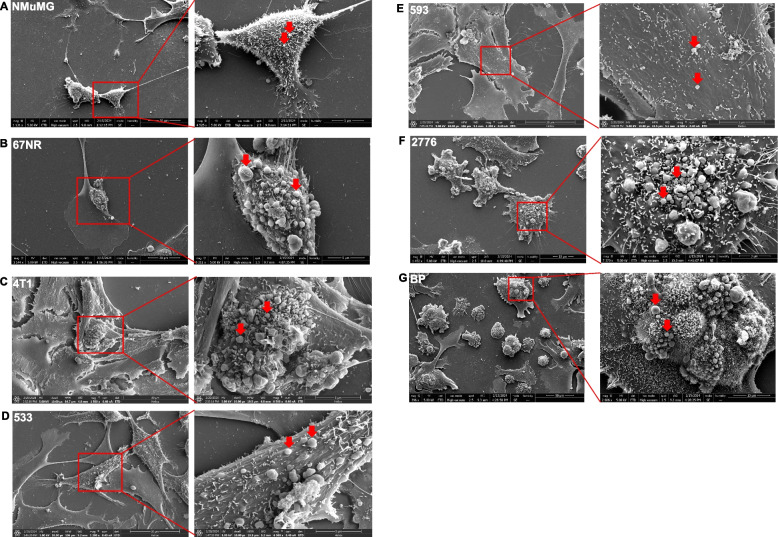
Scanning electron microscopy imaging of cell cultures displaying microvesicles-like structures lining the cell membranes. Representative SEM images showing microvilli and budding of EVs on their surface (red arrows) in (**A**) non-transformed NMuMG cells, **B** non-metastatic 67NR primary BC cells, **C** metastatic 4T1 parental cells, **D** lung-MBC 4T1-533 cells, **E** bone-MBC 4T1-593 cells, **F** liver-MBC 4T1-2776 cells and (**G**) brain-MBC 4T1-BP cells. The box insert shows spherical vesicles on the cell surface. Magnification = 6500X

We were interested in seeing not only the quantity of EVs emitted but also how the vesicles attach to the cells. We thus further characterized the cell surface topography using SEM and observed that the cell membrane is decorated with microvesicle-like structures reminiscent of EVs (Fig. 3). Metastatic cancer cells exhibited isolated globular shapes with varying sizes of EVs on their surface. These observations suggest that oncogenic transformation may play an important role in cellular morphology as well as in EV biogenesis. We next treated the cells with the non-competitive neutral sphingomyelinase (nSMase) inhibitor GW4869, known to suppress EV biogenesis [77, 78], to see the effect on these cell lines. Notably, 4T1, 4T1-2776 and 4T1-BP treated with GW4869 (10 μM and 20 μM) for 12 h showed a significant reduction in EV budding from the cell surface (Figure S4).

### Organotropic MBC cells released elevated levels of mutated EV-DNA and cfDNA

**Fig. 4:**
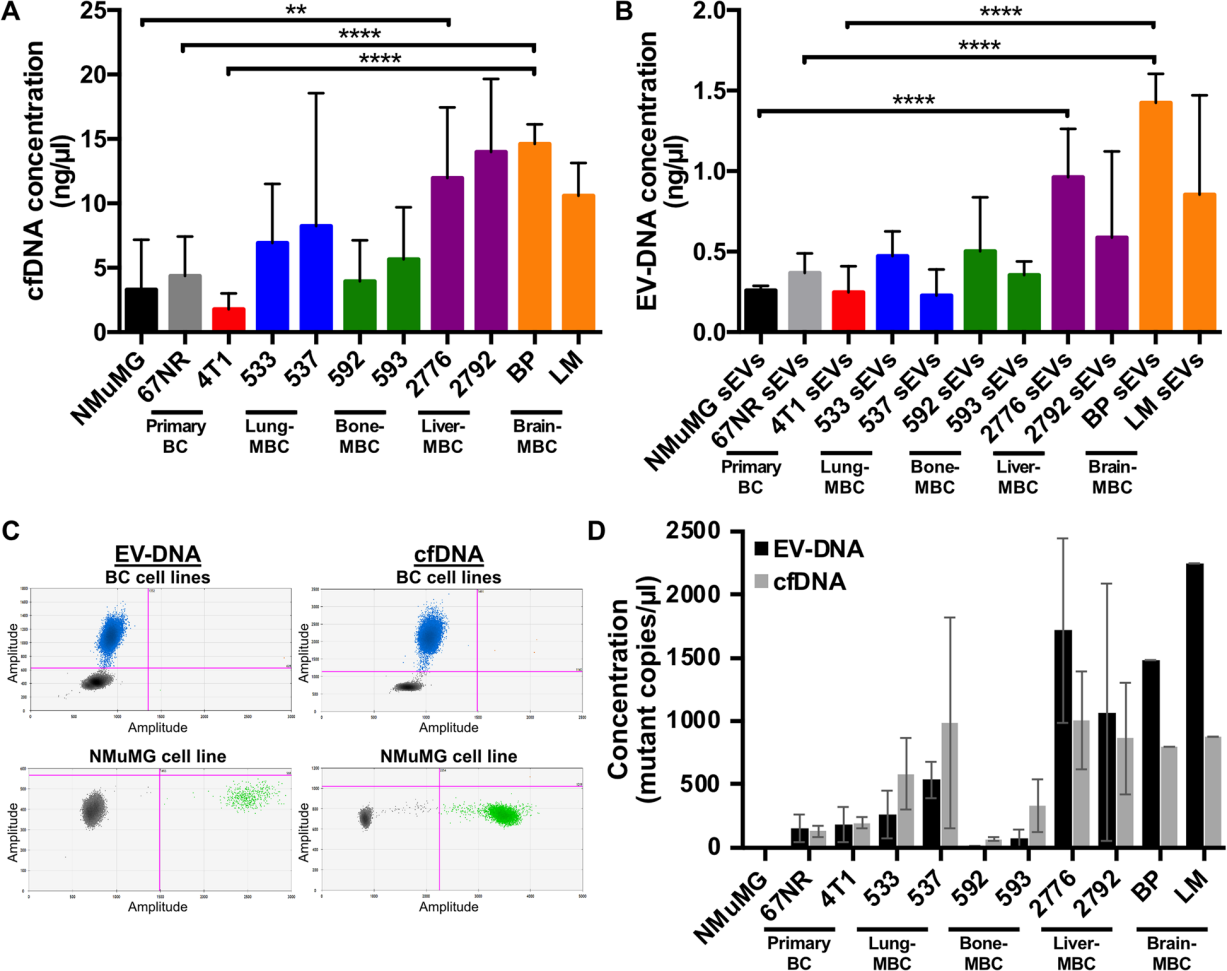
Analysis of cfDNA and EV-DNA isolated from primary BC, parental MBC and organotropic MBC cells. **A** cfDNA levels normalized to CCM volume. **B** EV-DNA levels normalized to total cell count. **C** Two-dimensional ddPCR plots representing the detection of the *Trp53* P31X mutation in EV-DNA and cfDNA isolated from the BC cell lines. Blue dots represent mutant-positive droplets; green dots represent wild type-positive droplets. **D** Concentration of mutant EV-DNA and cfDNA from BC and site-specific MBC cell lines. In A and B, data are presented as mean ± SEM (*n* = 3 independent experiments, **P: 0.0051 (NMuMG vs 4T1-2776), ***P: 0.0008 (NMuMG vs 4T1-2776), ****P < 0.0001). In D, data are presented as mean ± SD (*n* = 2 independent experiments)

We next investigated the presence of DNA cargo in the cell-derived EVs. Given the growing role of ctDNA as a cancer biomarker [79–81], we first determined whether we could detect cfDNA in the CCM. Indeed, we detected cfDNA in all cell lines by fluorometry, with variations in DNA concentrations across cell types (Fig. 4A). As EV-associated DNA has recently emerged as a novel biomarker [59], we next sought to determine whether the cfDNA detected was at least partially associated with EVs. Interestingly, EV-DNA was detected in all samples (Fig. 4B) and EV-DNA showed a positive correlation with cfDNA (R = 0.7862, *p* = 0.004) (Figure S5). The concentrations of both cfDNA and EV-DNA were consistently significantly higher in liver-MBC (4T1-2776) and brain-MBC (4T1-BP) cell lines when compared to their levels in non-transformed NMuMG, non-metastatic 67NR, and metastatic 4T1 (Fig. 4A-B).

While total cfDNA and EV-DNA in patients originate from various cell types throughout the body, the fraction attributed to cancer cells, known as ctDNA, is generally quantified using genomic alterations specific to the cancer cells, such as point mutations [82]. We therefore sought to assess the presence of mutant cfDNA in the 4T1 lineage cells, which carry a *Trp53* P31X mutation (human *TP53*), as confirmed by sanger sequencing (Figure S6). As anticipated, *Trp53* P31X mutant copies were specifically detected in cfDNA and EV-DNA of parental and site-specific MBC cells using ddPCR while only wild type copies were detected from non-transformed NMuMG cells (Fig. 4C-D, Figure S7). Mutant copies were more abundant in cfDNA and EV-DNA from lung-, liver-, and brain-MBC cell lines compared to non-transformed or primary BC cells, but less abundant in bone-MBC cells (Fig. 4D).

### EV-associated protein expression profiles clustered according to metastatic site

**Fig. 5:**
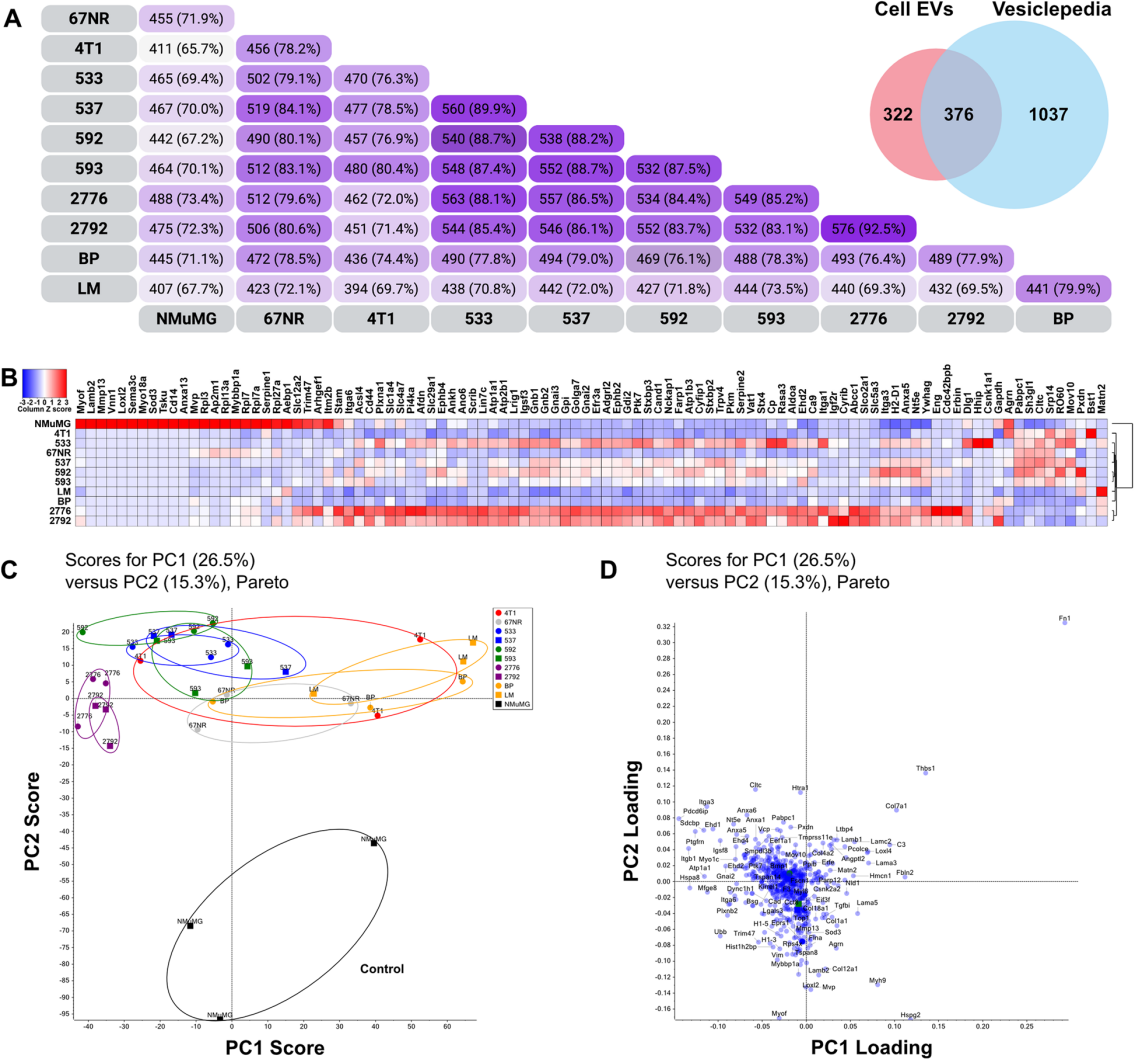
Proteomes of EVs derived from primary BC and site-specific MBC cell lines. **A** Venn diagram analyses. Sample datasets were compared for shared proteins between EVs isolated from the different cell lines. In the insert are shown samples datasets compared to EV protein cargo reported in the Vesiclepedia database (see Supplementary Table 1 for the full list of proteins, and Supplementary Table 2 for novel reported proteins). **B** Differential enrichment of EV-associated proteins derived from primary BC and MBC cell lines. The 100 most differentially abundant proteins in the cell-derived EVs identified by ANOVA (*p*-value < 0.05) are shown. (C and D) Scatter plot of the PCA results with the PC1 and PC2 scores assigned to each spectrum. This analysis includes 698 identified proteins (*n* = 3 independent EV preparations for each cell line). **C** 2D PCA scores plot of the 11 EV experimental groups. **D** PCA correlation loading plot. PC1 and PC2 represent the first and second principal components derived from PCA, respectively, capturing the directions of maximum and subsequent variance in the dataset. Data was normalized (Scaffold) and adjusted with Pareto scaling (MarkerView)

EVs contain a wide variety of proteins that contribute to biological processes and disease progression. To gain understanding of the functions of EVs in cancer metastasis, we analysed the proteomes of EVs isolated from primary BC, MBC and site-specific MBC cell lines in this organotropism model. Using a quantitative proteomic analysis, we identified 698 proteins of which 376 (54%) overlap with EV proteins previously reported in the Vesiclepedia database (Fig. 5A, Supplementary Table 1 and PRIDE repository ID: PXD055261). Further analysis enabled the identification of common (Table 1) and cell line-specific (Table 2) EV-associated proteins. Of the 376 shared proteins, many are known as typical markers of EVs (i.e., Annexins, Syntenin-1 (Sdcbp), TSG101, CD81, CD9, Flotillin, Alix (Pdcd6ip)). TSG101 and Alix are components of the endosomal sorting complex required for transport (ESCRT). Other adhesion and extracellular matrix (ECM) proteins such as fibronectin (FN1) and lactadherin (MFGE8) were identified, as well as transmembrane proteins including integrins. Linker histone H1-3 and core histones (H2 AC15, HIST1H2BP, H4 C1) were also found in all samples, denoting the presence of proteins associated with the nucleus (Table 1). In addition, we are reporting the addition of 322 novel proteins in EVs cargo, and most of them clustered in specific EV-derived tissues (Lung (68%), Liver (52%), Brain (65%)) (based on DAVID database analyses) (Fig. 5A, Supplementary Table 2).

**Table 1:** Common proteins identified in cell-derived EVs categorized by association with cellular localizations and classified by function. Includes 42 proteins consistently found across all three independent replicates of sites-specific MBC EVs, primary BC EVs, and non-transformed EVs

Identified Proteins	Accession #	Alternate ID	Class
**Plasma Membrane**			
Annexin A11	P97384	ANXA11	Transport
Annexin A2	P07356	ANXA2	Signal transduction
Annexin A3	O35639	ANXA3	Signal transduction
Annexin A5	P48036	ANXA5	Signal transduction
Clathrin heavy chain 1	Q68FD5	CLTC	Transport
Guanine nucleotide-binding protein G(I)/G(S)/G(T) subunit beta-1	P62874	GNB1	Signal transduction
Immunoglobulin superfamily member 8	Q8R366	IGSF8	Adhesion
Integrin alpha-3	Q62470	ITGA3	Adhesion
Integrin beta-1	P09055	ITGB1	Adhesion
Lactadherin	P21956	MFGE8	Adhesion
Moesin	P26041	MSN	Cytoskeleton
Ras-related C3 botulinum toxin substrate 1	P63001	RAC1	Cytoskeleton
Thrombospondin-1	P35441	THBS1	Adhesion
Transferrin receptor protein 1	Q62351	TFRC	Transport
**Cytoplasm**			
Actin, cytoplasmic 2	P63260	ACTG1	Cytoskeleton
Glyceraldehyde-3-phosphate dehydrogenase	P16858	GAPDH	Metabolism
Heat shock 70 kDa protein 1B	P17879	HSPA1B	Chaperone/Stress
Heat shock cognate 71 kDa protein	P63017	HSPA8	Chaperone/Stress
Heat shock protein HSP 90-beta	P11499	HSP90AB1	Chaperone/Stress
L-lactate dehydrogenase A chain	P06151	LDHA	Metabolism
Myosin-9	Q8VDD5	MYH9	Cytoskeleton
Polyadenylate-binding protein 1	P29341	PABPC1	RNA binding?
Polyubiquitin-B	P0CG49 (+1)	UBB	Metabolism
Programmed cell death 6-interacting protein	Q9WU78	PDCD6IP	Apoptosis
Prostaglandin F2 receptor negative regulator	Q9WV91	PTGFRN	Signal modulation
Putative helicase MOV-10	P23249	MOV10	RNA binding?
Syntenin-1	O08992	SDCBP	Cytoskeleton
Tubulin alpha-1B chain	P05213	TUBA1B	Cytoskeleton
Tumor susceptibility gene 101 protein	Q61187	TSG101	Transport
**Nucleus**			
Elongation factor 1-alpha 1	P10126	EEF1A1	Metabolism
Histone H1.3	P43277	H1-3	Chromatin associated
Histone H2A type 1-K	Q8CGP7	H2AC15	Chromatin associated
Histone H2B type 1-P	Q8CGP2	HIST1H2BP	Chromatin associated
Histone H4	P62806	H4C1	Chromatin associated
**Extracellular Matrix**			
Basement membrane-specific heparan sulfate proteoglycan core protein	Q05793	HSPG2	Adhesion
Collagen alpha-1(XVIII) chain	P39061	COL18A1	Extracellular
Collagen alpha-2(IV) chain	P08122	COL4A2	Extracellular
Extracellular matrix protein 1	Q61508	ECM1	Extracellular
Fibronectin	P11276	FN1	Extracellular
Fibulin-2	P37889	FBLN2	Adhesion
Lysyl oxidase homolog 4	Q924C6	LOXL4	Extracellular
Tubulointerstitial nephritis antigen-like	Q99JR5	TINAGL1	Extracellular

**Table 2:** Exclusive proteins identified in EVs from primary BC and site-specific MBC cell lines

Identified Proteins	Accession #	Alternate ID
**Breast cancer cell lines**		
ADP-ribosylation factor 3	P61205 (+ 1)	ARF3
Annexin A6	P14824	ANXA6
Bone marrow stromal antigen 2	Q8R2Q8	BST2
CD81 antigen	P35762	CD81
Nidogen-1	P10493	NID1
Peptidyl-prolyl cis–trans isomerase A	P17742	PPIA
Ras-related protein Rap-1b	Q99 JI6	RAP1B
Serine protease HTRA1	Q9R118	HTRA1
Solute carrier family 2, facilitated glucose transporter member 1	P17809	SLC2 A1
**Metastatic breast cancer cell line (4T1)**		
ADP-ribosyl cyclase/cyclic ADP-ribose hydrolase 2	Q64277	BST1
**Non-metastatic breast cancer cell line (67NR)**		
FACT complex subunit SPT16	Q920B9	SUPT16H
**All site-specific metastatic breast cancer cell lines**		
Bone morphogenetic protein 1	P98063	BMP1
CD9 antigen	P40240	CD9
Eukaryotic translation initiation factor 5 A-1	P63242	EIF5 A
Peptidyl-prolyl cis–trans isomerase B	P24369	PPIB
Phosphoglycerate mutase 1	Q9DBJ1	PGAM1
Protein tweety homolog 3	Q6P5 F7	TTYH3
Rho GDP-dissociation inhibitor 1	Q99PT1	ARHGDIA
Vacuolar protein sorting-associated protein 37B	Q8R0 J7	VPS37B
**Lung-metastatic breast cancer cell lines**		
EGF-containing fibulin-like extracellular matrix protein 1	Q8BPB5	EFEMP1
EGF-containing fibulin-like extracellular matrix protein 2	Q9 WVJ9	EFEMP2
Sulfhydryl oxidase 1	Q8BND5	QSOX1
**Liver-metastatic breast cancer cell lines**		
1-phosphatidylinositol 4,5-bisphosphate phosphodiesterase delta-3	Q8 K2 J0	PLCD3
26S proteasome regulatory subunit 10B	P62334	PSMC6
Actin-binding protein WASF2	Q8BH43	WASF2
ATP-dependent 6-phosphofructokinase, liver type	P12382	PFKL
Casein kinase II subunit alpha	Q60737	CSNK2 A1
Coiled-coil and C2 domain-containing protein 1B	Q8BRN9	CC2D1B
Endoglin	Q63961	ENG
Integrin alpha-7	Q61738	ITGA7
Leucyl-cystinyl aminopeptidase	Q8 C129	LNPEP
Metalloreductase STEAP3	Q8 CI59	STEAP3
Multidrug resistance-associated protein 1	O35379	ABCC1
Out at first protein homolog	Q8QZR4	OAF
Phosphatidylinositol 4-kinase alpha	E9Q3L2	PI4 KA
Protein-arginine deiminase type-4	Q9Z183	PADI4
Scavenger receptor class B member 1	Q61009	SCARB1
Serine/threonine-protein phosphatase 2 A catalytic subunit α isoform	P63330	PPP2 CA
Solute carrier family 12 member 7	Q9 WVL3	SLC12 A7
Solute carrier organic anion transporter family member 2 A1	Q9EPT5	SLCO2 A1
Sorting nexin-18	Q91ZR2	SNX18
Unconventional myosin-Ixb	Q9QY06	MYO9B
V-type proton ATPase catalytic subunit A	P50516	ATP6 V1 A
V-type proton ATPase subunit d 1	P51863	ATP6 V0D1
Vacuolar protein sorting-associated protein 35	Q9EQH3	VPS35
Vacuolar-sorting protein SNF8	Q9 CZ28	SNF8
**Brain-metastatic breast cancer cell lines**		
Laminin subunit alpha-3	Q61789	LAMA3

Interestingly, analyses of the differentially enriched EV-associated proteins revealed that those from NMuMG cells clustered differently from those isolated from cancerous cells, and organotropic cell lines clustered differently from each other (Fig. 5B). In addition, distinct clustering based on protein expression profiles was observed through principal component analyses (PCA), revealing inherent differences between EVs from primary BC and site-specific MBC cell line sources, with non-transformed cells widely separated as expected (Fig. 5C-D). The PCA also highlighted proteins contributing significantly to these separations. Notably, EVs isolated from the same organotropic cells shared high numbers of proteins (i.e., lung (86.7%), bone (87.4%), liver (92.5%) and brain (75.4%)), and while EVs derived from cancer cells shared high number of proteins (65.4–79.5%), they shared a smaller number of proteins with the non-transformed cells (57.4–69.6%). Together, this offers insights into potential biomarkers or functional distinctions among EVs based on cellular origins (Fig. 5C-D). Further confirmation with stain-free gels showed a distinct EV protein profile of non-transformed NMuMG compared to primary BC- and metastatic BC-derived EVs (Figure S8). This suggests that the protein cargo of EVs might represent a tool for MBC clustering.

### BC cell-derived EVs carry proteins involved in the oncogenic and metastatic processes

**Fig. 6:**
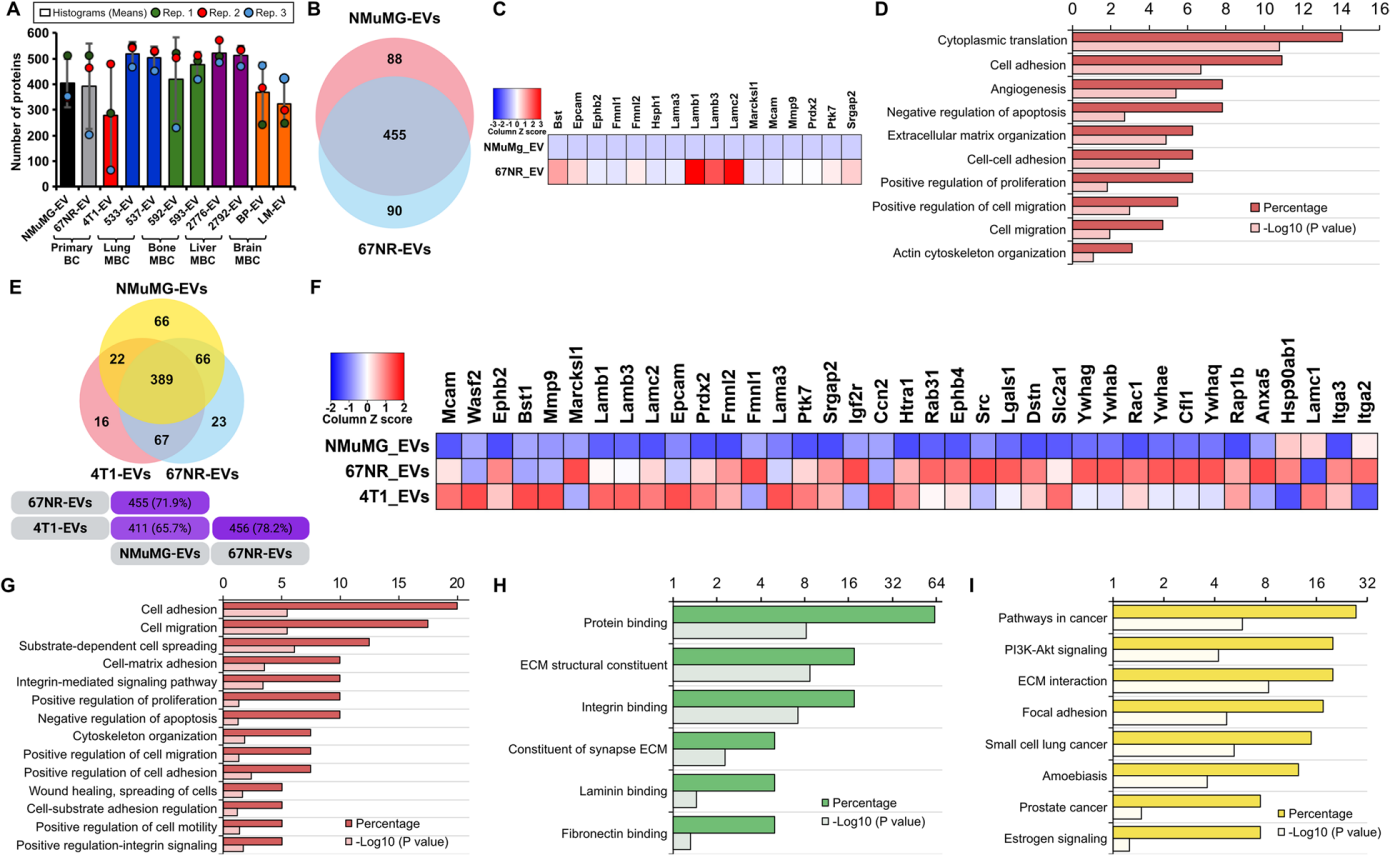
BC EVs are enriched in proteins involved in oncogenic and metastatic processes. **A** Total protein counts from EVs isolated from each cell line. The analysis includes a total of 698 identified proteins. Data was retrieved from normalized TSC. Data are presented as mean ± SD (*n* = 3 independent experiments (Histograms)). Note that dots represented values for the three replicates. **B** Venn diagram analyses. Samples datasets were compared for shared proteins between EVs isolated from NMuMG and 67NR cell lines (see Supplementary Table 3 for the list of proteins). **C** Heatmap chart depicting the relative expression levels of proteins linked to tumorigenesis. Note that both datasets clustered differently one from the other. **D** GO classification of proteomic data for the differentially expressed proteins. The most enriched categories in Biological Process are shown (see Supplementary Table 4 for the list of proteins). **E** Venn diagram analyses. Samples datasets were compared for shared proteins between EVs isolated from non-malignant (NMuMG) and cancerous (67NR and 4T1) cells (see Supplementary Table 5 for the list of proteins). **F** Heatmap chart depicting the relative expression levels of proteins linked to tumorigenesis. Note that datasets clustered differently one from one other. **G**-**I** GO classification of proteomic data for the differentially expressed proteins. The most enriched categories in (**G**) Biological Process, **H** Molecular Function and (**I**) KEGG are shown (see Supplementary Table 4 for the list of proteins)

A diversity of proteins was found in EVs from different cell lines with consistency across replicates. Lower protein abundance was detected in EVs from 4T1 parental and brain-tropic cells compared to the other cell lines (Fig. 6A). Among the 698 identified proteins from three independent experiments, an average of 404 proteins was found in EVs from non-transformed NMuMG cells. In contrast, EVs from primary BC cell lines contained averages of 393 proteins (67NR) and 278 proteins (4T1). For lung-MBC cell lines, average protein counts were 520 (4T1-533) and 504 (4T1-537). In bone-MBC cell lines, 419 (4T1-592) and 475 proteins (4T1-593) were identified. Liver-MBC cell-derived EVs contained on average 523 (4T1-2776) and 514 proteins (4T1-2792), while brain-MBC cell-derived EVs had on average 368 (4T1-BP) and 323 proteins (4T1-LM) (Fig. 6A).

We used this proteomic dataset to perform extensive comparisons of EV protein cargo. First, we focused on protein differential expression between non-transformed NMuMG and cancerous 67NR cells to determine factors involved in carcinogenesis (Fig. 6B and Supplementary Table 3). This analysis allowed to determine the presence in the 67NR-EV samples of proteins involved in angiogenesis (i.e., EPHB2, COL8 A1, MCAM), regulation of proliferation (MARCKSl1, BST1, EPCAM, LAMC2, EPHB2), negative regulation of apoptosis (HSPH1, PRDX2), cell migration (PTK7, FMNL1, FMNL2, LAMB1, LAMA3, LAMB3, LAMC2, MMP9, EPHB2, MCAM), and cell adhesion (COL8 A1, LAMB1, LAMA3, LAMB3, LAMC2, MCAM, SRGAP2), that are all hallmarks of malignant transformation (Fig. 6C-D and Supplementary Table 4). Afterwards, we included in the comparisons proteins isolated from the MBC 4T1 cells. In these analyses, we merged both 67NR-EV and 4T1-EV datasets and compared them to NMuMG-EV dataset (Fig. 6E and Supplementary Table 5). In these analyses, we determined the presence in the malignant cell-EVs of additional highly expressed proteins involved in angiogenesis (i.e., EPHB4, WASF2, CCN2), regulation of proliferation (LAMA5, ITGA2, RHOA, SRC, MMP9, CNN2, YWHAG, YWHAB, YWHAE, YWHAQ), regulation of apoptosis (EPCAM, HSP90 AB1, HTRA1, SRC, ANXA5, CCN2, IGF2R, LGALS1, MMP9), cell migration (RAC1, SRC, CCN2, LAMB1, LAMA5, LAMB3, LAMC1, MMP9, RHOA, WASF2, CFL1, DSTN, MYOC1C), and cell adhesion (LAMB1, LAMB3, RHOA, RAP1B, RAC1, EPHB4, PTK7, RAC1, SRC, CCN2, ITGA2, ITGA3, LAMC1, LAMC2). In addition, we found that many highly expressed proteins are implicated in cellular binding (ephrin receptors, integrins, constituent of ECM) and oncogenesis (AGRN, NPNT, RAC1, HSP90AB1, RAB22 A, RAB31, SRC, ITGA2, ITGA3, LAMB1, LAMA3, LAMB3, LAMC1, LAMC2, MMP9, SLC2 A1, RAP1B) (Fig. 6F-I and Supplementary Table 4). Together, these data indicate that EV protein cargo inform on the identity of shedding cell types and cellular functions they might regulate.

**Fig. 7:**
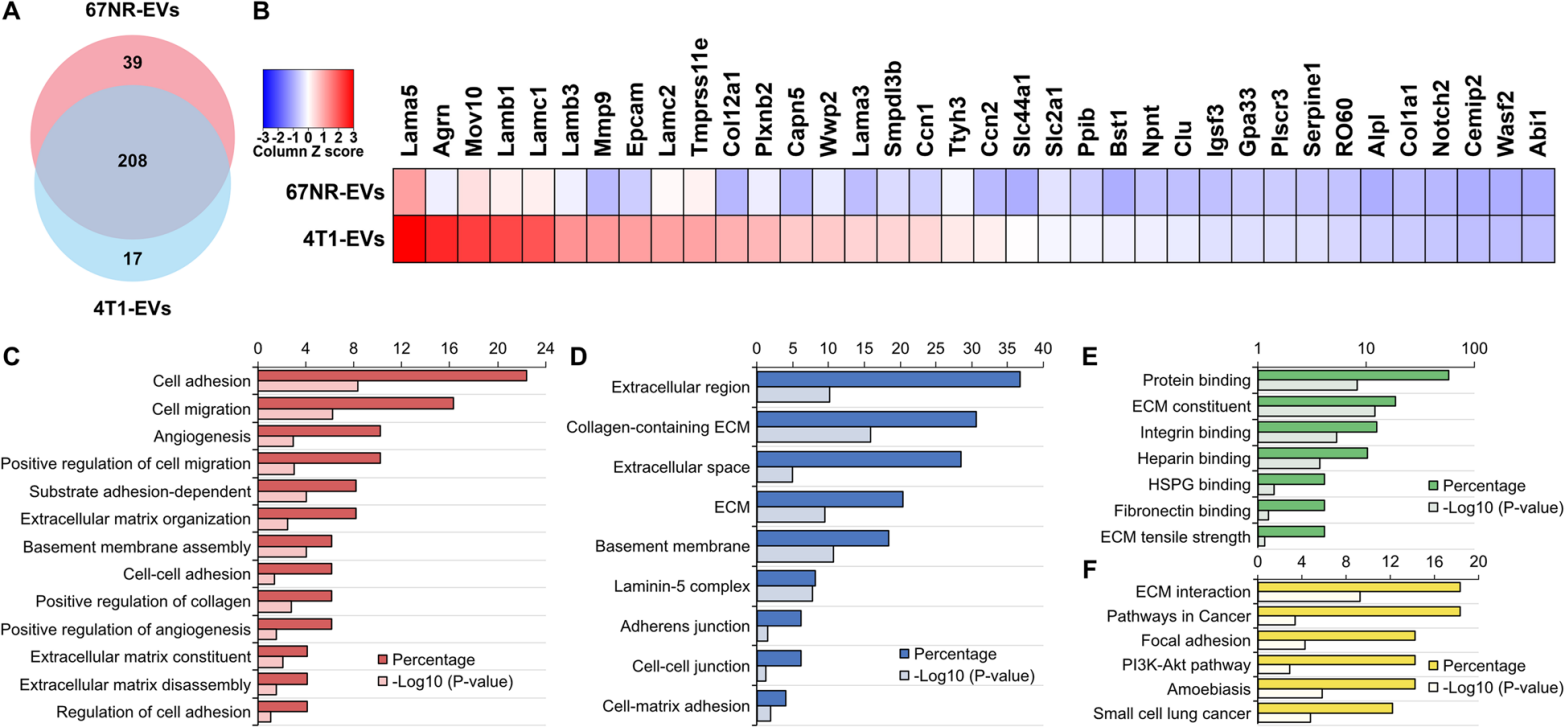
Metastatic BC EVs are enriched in proteins involved in metastasis regulation and metastatic niche organization. **A** Venn diagram analyses. Sample datasets were compared for protein differential expression between EVs isolated from 67NR and 4T1 cell lines (see Supplementary Table 6 for the list of proteins). **B** Heatmap chart depicting the relative expression levels of proteins linked to metastasis. Note that both datasets clustered differently one from the other. **C**-**F** GO classification of proteomic data for the differentially expressed proteins. The most enriched categories in (**C**) Biological Process, **D** Cellular Component, **E** Molecular Function and (**F**) KEGG are shown (see Supplementary Table 7 for the list of proteins)

We then mined our data by comparing 67NR-EV and 4T1-EV datasets and focusing on proteins involved in metastasis regulation and metastatic niche environment modulation (Fig. 7 and Supplementary Table 6). We found proteins exclusively present or highly expressed in 4T1-EVs (Fig. 7A-B). Gene ontology scanning of these proteins displayed the presence of many factors having metastatic-inducing potentials and that clustered in categories related to hallmark of metastasis (cell adhesion, cell migration, regulation of angiogenesis and metastatic microenvironment organization) (Fig. 7C-F). Specifically, some proteins are involved in angiogenesis disruption and subsequent facilitation of tumor cell metastasis (i.e., Eng). Other proteins are known inducers of epithelial to mesenchymal transition (EMT) (i.e. Laminins) or cancer cell spreading (i.e., CCN2, NPNT, WASF2, SERPINE, MAP4K4). Notably, the majority of these proteins are involved in pre-metastatic niche formation (i.e., Collagens, Laminins, MMP9, NPNT, CCN2, ENG, SERPINE1, AGRN, PLXNB2, ALPL, GLG1, PTPRF, PTPRS) (Supplementary Table 7).

**Fig. 8:**
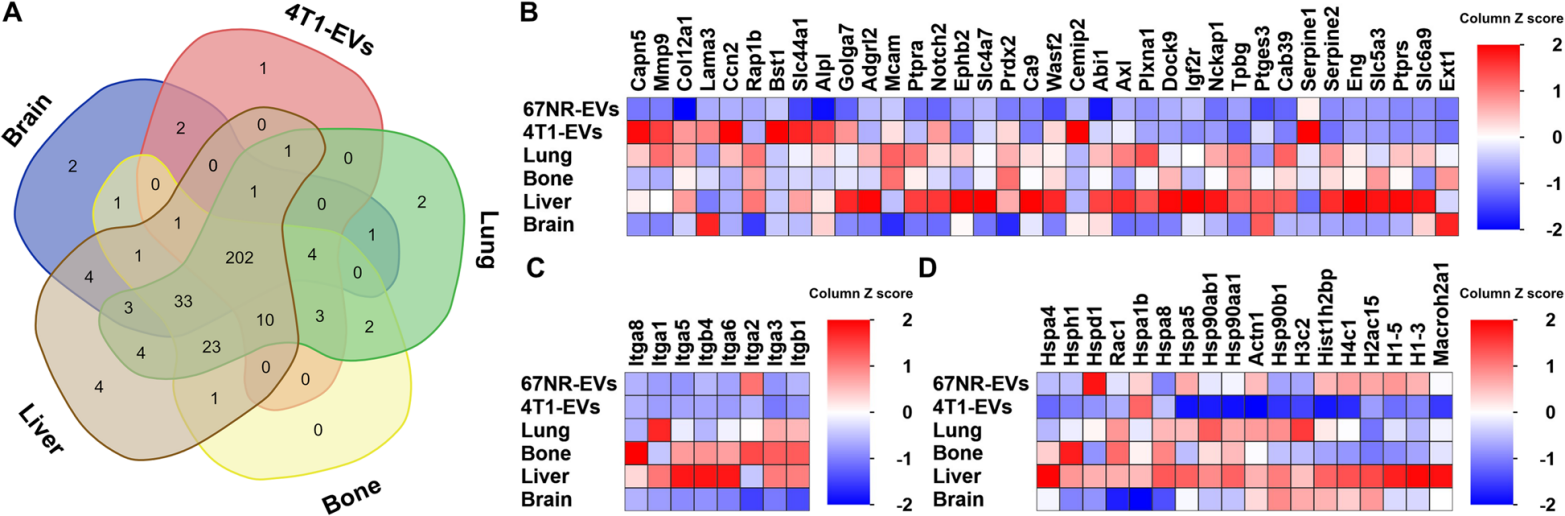
Metastatic BC EVs carry proteins involved in organotropic homing. **A** Venn diagram analyses. Sample datasets were compared for protein differential expression between EVs isolated from MBC cell lines (see Supplementary Table 9 for the list of proteins). The graph was drawn in https://bioinformatics.psb.ugent.be/webtools/Venn/. **B** Heatmap chart depicting differentially expressed proteins between 67NR-EVs and MBC-EVs. **C**-**E** Heatmap charts showing differentially expressed proteins between 67NR-EVs and MBC-EVs in the categories of (**C**) integrins, and (**D**) DNA-binding proteins

As one of our goals was to unravel determinants of BC metastatic organotropism, we scanned our proteomic data outputs for putative factors that might have a role in this process (Fig. 5A, Fig. 8 and Supplementary Table 8). First, we determined the distribution pattern of the highly expressed proteins in BC samples. We found that some proteins were exclusively present in lung (*n* = 2), liver (*n* = 4) and brain (*n* = 2) organotropic BC cell-derived EVs (Fig. 8A, Table 3 and Supplementary Table 9). Of these exclusively expressed proteins, only 2 (WASL and LRP5) are involved in metastatic processes and none were reported to be involved in specific tissue-targeted cell homing or organotropic cancer metastasis. More studies are warranted to determine whether some might be involved in these processes.

**Table 3:** List of proteins exclusively expressed in single organotropic MBC cell-derived EVs

4T1	Lung	Bone	Liver	Brain
BST1	HHIP, WASL		LRP5, ARHGEF1, TMEM106B, NOP56	LAMB2, SOD3

When we mined the differentially expressed protein datasets, we found that some displayed organotropism-inducing potential (Fig. 8B). Specifically, overexpression of the cellular communication network factor 2 (CCN2/CTGF) was associated with BC metastasis to bone. It contributes to bone metastasis by converting low-metastatic BC cells to high-metastatic ones in collaboration with other factors [83, 84]. Also, MMP9 was enriched in 4T1 parental, lung-, bone-, and liver-MBC cell-EVs. MMP9 was reported to enhance cancer metastasis to lung and liver through collagen degradation at metastatic niches [85, 86]. Serpins were reported to be involved in spontaneous brain metastasis [87, 88]. In our analyses, we found that serpins were highly expressed in MBC cell-EVs (Fig. 8B). Integrins, that were described as mediators of cancer organotropism [43], are highly expressed in at least one of the MBC cell-EVs. Notably, several integrins displayed higher expression levels in tissue-specific MBC cell-EVs, except for the brain MBC cell-EVs (Fig. 8C). Other upregulated proteins have been previously linked to cancer cellhomeostasis including the molecular chaperon proteins (i.e., DNA binding proteins and heat shock proteins (HSP)). Specifically, of the DNA binding proteins, the majority of histones are highly expressed in tissue-specific MBC cell-derived EVs (Fig. 8D). In addition, HSP were highly present in tissue-specific MBC cell-EVs (Fig. 8D).

Other proteins were also found exclusively or highly expressed in MBC cell-derived EVs. We noted the enrichment of 10 distinct types of annexins. Notably, ANXA3, A5, A6 and A11 were significantly enriched in lung-MBC, bone-MBC, and liver-MBC cell-EVs, while ANXA4 was enriched in lung-MBC and liver-MBC EVs. CD44 were both enriched in, bone-MBC and liver-MBC cell-derived EVs. Moreover, we noticed the significant enrichment of 5 types of collagens in all site-specific MBC cell-EVs (lung, bone, liver, brain) compared to parental 4T1 EVs. In addition, the expression of various glycolytic enzymes (ENO1, ENO3, LDHA, ALDOA) was also predominant in liver and lung MBC cell-EVs. Signal transduction proteins such as YWHA proteins (i.e., YWHAB, YWHAE, YWHAG, YWHAH, YWHAQ, YWHAZ) were highly enriched in lung, bone, and liver MBC cell-EVs (Supplementary Table 8). Together, these analyses revealed that the protein cargos could be differentially enriched in the EVs of cells with varying metastatic potential. This opens the way to use this information for disease staging.

## Discussion

Gaps remain in our understanding of the mechanisms that govern preferential metastasis of cancer cells to specific organs. Consequently, there is a lack of reliable and clinically valuable biomarkers for metastasis, largely due to the challenges posed by the scarcity of tissue samples from metastatic sites and the availability of appropriate models for site-specific metastasis. To address this issue, EVs present a promising avenue for research optic. In this study, we used a unique cell model of BC organotropism to various organs and characterized the EV molecular content with the goal of uncovering important mediators of metastasis that could serve as biomarkers and/or therapeutic targets [26].

We investigated the amount and nature of released DNA by exploring both EV-DNA and cfDNA. The quantification of cfDNA has shown prognostic and predictive value in many cancers [89]. Its detection enables early identification, lesion classification, assessment of mutational load, and real-time monitoring of disease response to treatment, providing valuable insights into the condition of tumors and cellular systems [90–92]. Specifically, plasma DNA concentration is significantly higher in BC patients compared to healthy women [57, 79]. Herein, we report significantly higher cfDNA levels in BC cell lines compared to normal cells, with liver- and brain-MBC cell lines showing the highest concentrations. Moreover, ctDNA has been used to detect metastatic disease in BC patients and predict genetic events associated with metastatic relapse more accurately than sequencing on primary BC tissue [93]. A significant association exists between specific genetic alterations and survival outcomes, with *TP53* mutations linked to poorer survival in human MBC [24]. We detected *Trp53* mutated cfDNA across all analyzed murine BC cell lines with highest levels released by lung-, liver-, and brain-MBC cell lines, confirming that cfDNA mutational status for MBC can be achieved using this approach. However, these differences warrant further investigation to determine their biological significance and value for screening purposes.

Growing evidence linked EV-DNA levels in liquid biopsies to diseases status including cancer [94, 95]. We recently developed a database (i.e., EV-ADD (www.evdnadatabase.com)) to gather these efforts [60]. EV-DNA has utility in the detection of mutations that reflect the genetic landscape and heterogeneity of the tumor of origin [53, 54, 96]. In the context of BC, EV-DNA has been identified in various subpopulations of EVs [97]. Herein, we detected mutant *Trp53* EV-DNA across all cancer cell lines that allowed to distinguish non-transformed from cancer states. This highlights the utility of quantifying such genetic alterations for the follow-up of MBC patients [98, 99]. Although EVs provide a shield to their cargo [100, 101], we reported a strong correlation between the levels of mutated cfDNA and EV-DNA. These findings are limited to a murine in vitro model and therefore require extensive confirmation using in vivo and human clinical samples. The identification of organ-specific EV proteins and DNA mutations opens new possibilities for non-invasive diagnostics using liquid biopsies. For example, mutant *Trp53* EV-DNA could serve as a biomarker for early detection of metastatic progression in BC patients. Furthermore, targeting integrins enriched in metastatic EVs could provide novel therapeutic strategies tailored to specific metastatic sites.

Using MS proteomic analyses, we detected significant enrichment of proteins involved in cancer progression and organotropism that are unique to specific metastatic sites. Specifically, we found that integrins were enriched and differentially expressed in EVs according to metastatic site. The ITGαVβV complex plays a role in various biological functions (i.e., cell migration, invasion, and interaction with the ECM) [102, 103]. Notably, we and others reported that ITGαVβV-enriched cancer EVs triggered changes in recipient cells and favored liver metastasis organotropism [43]. [104]. Also, we identified significant enrichment of key proteins associated with the ITGαVβV (i.e., ITGA3, ITGB1) in EVs from lung-, bone-, and liver-MBC cell lines, which aligns with their role in mammary tumorigenesis [ 43, 45, 105, 106, 107, 108–110].

CD44 are immune evasion proteins which expression was linked to BC metastasis and poor prognosis [111–113]. We detected both proteins in liver-, lung-, and bone-MBC cell-EVs. This suggests that MBC-EVs may convoy immune escape to MBC cells. Moreover, we found that a panel of annexins are enriched in site-specific MBC EVs in accordance with their reported roles in cancer aggressiveness and chemoresistance [114, 115].

Cancer EV proteins are essential for metastatic niche ECM remodeling and successful cancer cell homing. We and others reported that metalloproteinase (MMP)-dependent collagen degradation underlay metastasis progression and patients’ poor outcome [85, 116–120]. Our finding that only MMP9 was enriched in MBC-EVs positions it as major inducer of ECM organization [121].

**Fig. 9:**
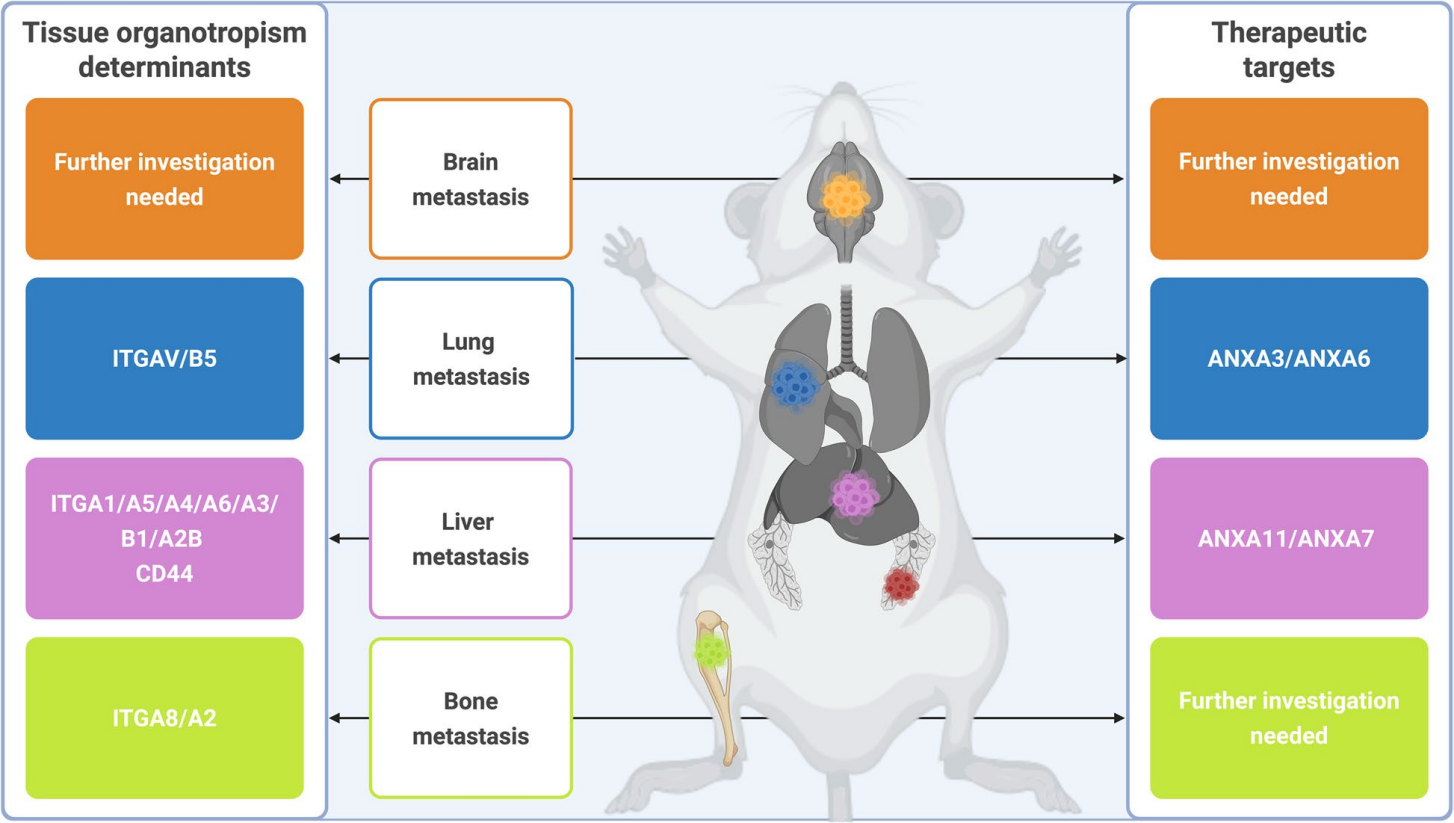
Overview of metastatic BC organotropism determinants and potential therapeutic targets. EV-associated proteins were differentially enriched in metastatic BC cells according to organotropism. Integrins (e.g., ITGAV/B5 for lung metastases, ITGA8/A2 for bone metastases) and annexins (e.g. ANXA3/ANXA6 in lung metastases, ANXA11/ANXA7 in liver metastases) were identified as potential biomarkers and therapeutic targets. These findings suggest that EV cargo could inform disease staging and patient stratification while providing insights into tissue-specific therapeutic strategies. Created in BioRender. Burnier, J. (2025) https://BioRender.com/nnnamw1

In summary, these proteomic analyses revealed that the protein cargos could be differentially enriched in EVs of cells with varying metastatic potential. This opens the way to use this information for disease staging, and for patients’ stratification and care. Based on our findings, we can draw a picture to classify organotropic MBC based on EV marker signatures, and to determine markers that can represent therapeutic targets in tissue-specific MBC settings (Fig. 9). More precisely, as summarized in Fig. 9, our findings highlight the differential roles of integrins and annexins in shaping the metastatic preferences of BC cells. These proteins not only serve as potential biomarkers but also raise important questions about their mechanistic roles in pre-metastatic niche formation and immune evasion. For example, the enrichment of CD44 in EVs derived from liver-, lung-, and bone-metastatic cells suggests a dual role in promoting immune escape and enhancing metastatic colonization. We propose that ITGAV/B5, ITGA8/A2, and ITGA1/A5/A4/A6/A3/B1/A2B and CD44 might specify lung-, bone- and liver-BC metastasis, respectively (Fig. 9). On the other hand, we suggest that drug combinations targeting ANXA3/ANXA6, and ANXA11/ANXA7 might be useful against lung- and liver-metastatic diseases, respectively. Future studies should focus on validating these targets in clinical samples and exploring their therapeutic potential through drug combinations targeting ANXA11/ANXA7 or integrin inhibitors. In this optic, functional and in vivo studies are warranted to clarify these assumptions. In addition, further studies should be carried out to demonstrate the molecular and cellular mechanism by which these metastasis-specific proteins contribute to MBC, which may pave the way for biomarker discovery.

The use of EVs to monitor and predict therapeutic response in MBC patients is evolving. In our study, we isolated small EVs from CCM using ultracentrifugation, acknowledging its limitations—including the time-consuming process and potential co-isolation of non-vesicular particles [122–125]. Although current technologies lack the sensitivity and specificity to fully distinguish pure EVs [122–125], our approach lays a solid foundation for EV-based biomarker discovery. These findings support future clinical translation, where optimized EV isolation methods could enhance biomarker validation in patients with MBC.

## Conclusion

Our study demonstrates that EVs from BC cells may harbor significant potential in various applications. By characterizing the EV cargo from organotropic MBC cell lines, we identified specific proteins, such as integrins, that are differentially enriched according to metastatic sites. Additionally, we demonstrated that EV-DNA mirrors the mutational landscape of cancer cells, including the detection of *Trp53* mutations, which could serve as a non-invasive biomarker for disease progression. We found that EVs derived from BC cells selectively enrich metastatic factors and signaling components, shedding light on crucial regulatory networks within the tumor microenvironment. These findings provide valuable insights into the molecular mechanisms underlying organ-specific metastasis and offer a framework for developing liquid biopsy-based diagnostics and novel targeted therapies. Moving forward, efforts should focus on validating these protein signatures and genetic markers in patient cohorts to assess their diagnostic and prognostic utility. Functional studies are also warranted to explore how EV cargo modulates the tumor microenvironment and promotes metastasis. By bridging basic research with clinical applications, this work lays the foundation for innovative approaches to improve the diagnosis and treatment of metastatic breast cancer.

## Supplementary Information

Supplementary Material 1.

Supplementary Material 2.

Supplementary Material 3.

Supplementary Material 4.

Supplementary Material 5.

Supplementary Material 6.

Supplementary Material 7.

Supplementary Material 8.

Supplementary Material 9.

Supplementary Material 10.

## Data Availability

The mass spectrometry proteomics data have been deposited to the ProteomeXchange Consortium via the PRIDE partner repository [126] with the dataset identifier PXD055261.
