# Supplementary material for: Characterization of extracellular vesicle-associated DNA and proteins derived from organotropic metastatic breast cancer cells
Source: J Exp Clin Cancer Res. 2025 May 23;44:157. doi: 10.1186/s13046-025-03418-3 (PMC12100931; doi:10.1186/s13046-025-03418-3)
Supplement: Supplementary file 1 — Supplementary Material 1. [file 13046_2025_3418_MOESM1_ESM.docx]

**Supplementary Figures**

**Figure S1: Cell cultures of murine cell lines.** Phase contrast images were taken on live cells. Magnification: 20x.


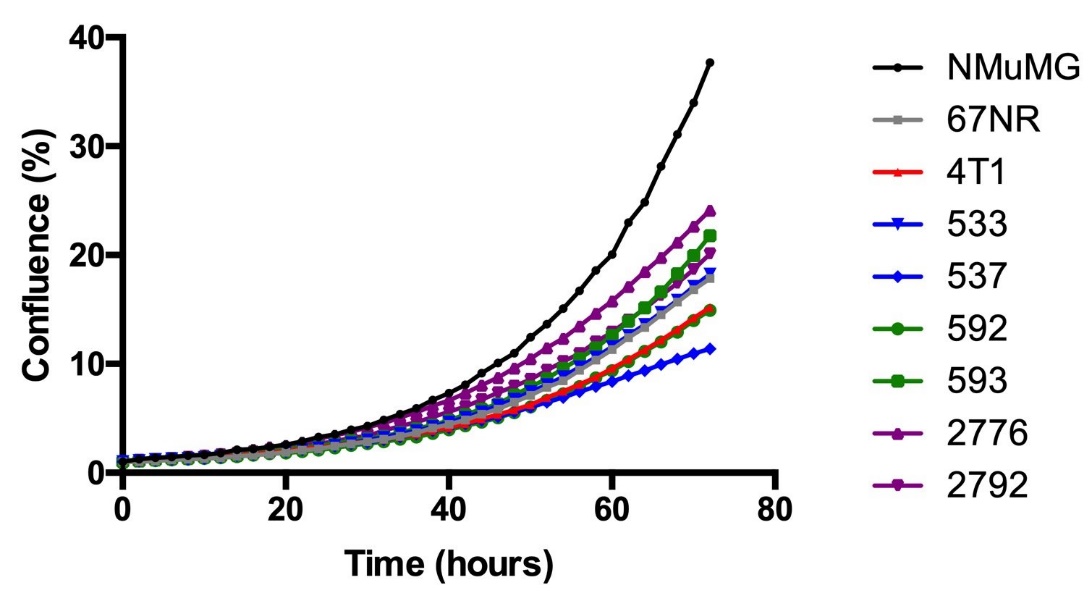


**Figure S2: Cell growth quantified by live cell imaging (Incucyte).** Cells were cultured for 72h.


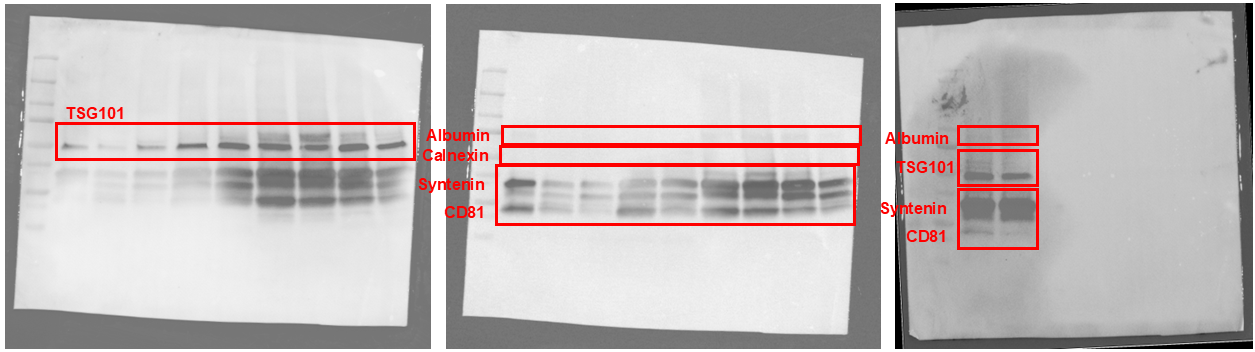


**Figure S3. Uncropped WB membranes (related to Figure 2E).**

**Figure S4: Scanning electron microscopy images of (A) 4T1, (B) 2776, and (C) BP cells untreated (top panels) and treated with GW4869 at 10 μM (middle panels) and 20 μM (bottom panels).** Scale bar: 10 μm.


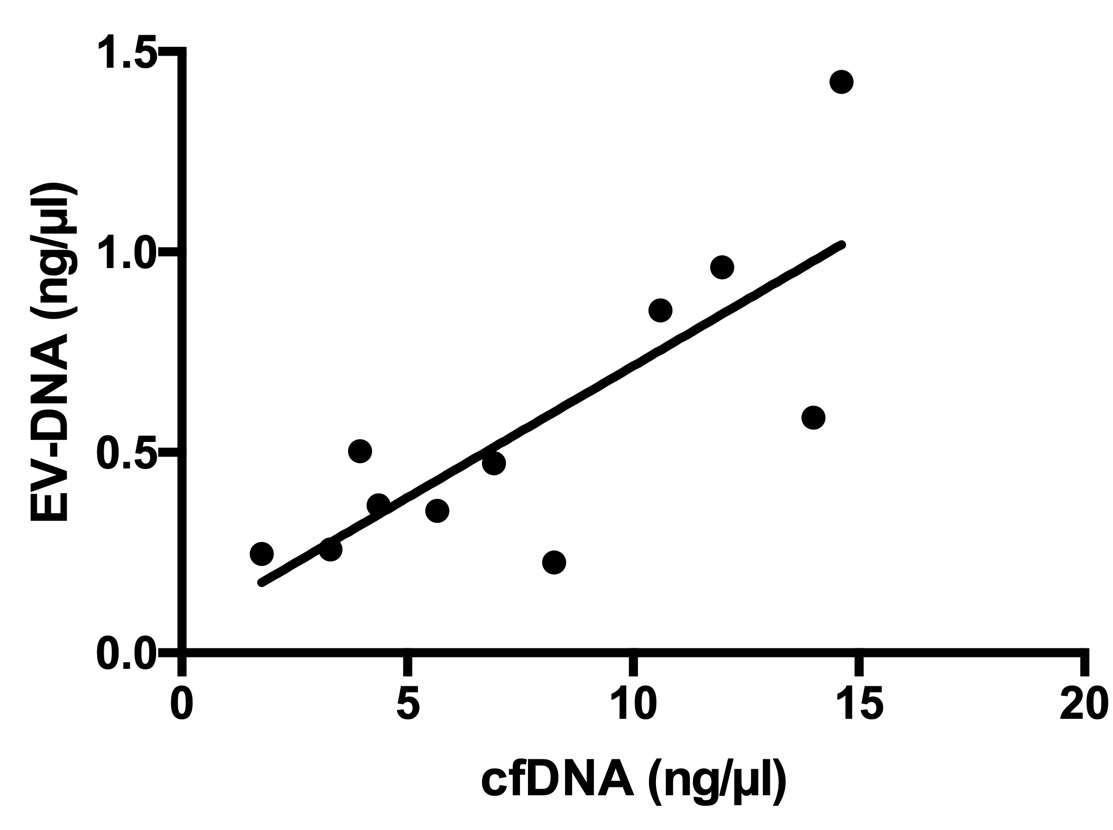


**Figure S5: Correlation of regression of cfDNA and EV-DNA.** R squared: 0.6181; R: 0.7862; 95% confidence interval; P-value ** (two-tailed): 0.0041. Pearson correlation coefficients. Number of values: 11 (cell lines, average of independent replicates). Equation: Y = 0.06564*X + 0.05913.

**Figure S6:** **Detection of mutant *Trp53* through Sanger Sequencing**. The electropherogram of the 4T1 sample shows one adenine nucleotide insertion in a homozygous manner resulting in Trp53X p.(P31Argfs*27). This frame shift mutation leads to stop codon at 58 and resulted in truncated Trp53 protein.

**Figure S7: Detection of tumor-suppressor gene mutation signature in cfDNA and EV-DNA using ddPCR.** (A) 1D-Amplitude plots of a ddPCR assay to detect the *Trp53* P31X gene mutation in EV-DNA. (B) 1D-Amplitude plots of a ddPCR assay to detect the *Trp53* P31X gene mutation in cfDNA. (C) 2D-plots of ddPCR results for EV-DNA (D) 2D-plot of ddPCR results for cfDNA. The green dots represent wild type-positive droplets.

**Figure S8: Stain free gel of 20 μg of 11 EV samples separated by SDS-PAGE gradient (4% −12%).**Note that 4T1, 67NR, lung-metastatic (4T1-533, 4T1-537) and bone-metastatic EVs (4T1-592, 4T1-593) have distinct bands (arrows) compared to non-transformed NMuMG EVs, liver-metastatic (4T1-2776, 4T1-2792) and brain-metastatic (4T1-BP, 4T1-LM) EVs. Gels are cropped to better highlight the differences.
